# Identification of a Ribosomal Protein RpsB as a Surface-Exposed Protein and Adhesin of* Rickettsia heilongjiangensis*

**DOI:** 10.1155/2019/9297129

**Published:** 2019-07-09

**Authors:** Yong Qi, Jixian Rao, Wanpeng Shen, Jiameng Li, Wenwen Zeng, Shulong Zheng, Suyun Liu, Yuping Li, Bijun Wang, Fan Wu, Yuchao Li, Yuexi Li

**Affiliations:** ^1^Huadong Research Institute for Medicine and Biotechniques, Nanjing 210002, China; ^2^College of Life Science and Technology, China Pharmaceutical University, Nanjing, Jiangsu, 210009, China; ^3^School of Public Health, Nanjing Medical University, Nanjing, Jiangsu, 210002, China

## Abstract

*Rickettsia heilongjiangensis* is an obligate intracellular bacterium that is responsible for far-eastern spotted fever. Surface-exposed proteins (SEPs) play important roles in its pathogenesis. Previous work identified a ribosomal protein RpsB as an SEP by biotin-avidin affinity, a seroreactive antigen, and a diagnostic candidate protein, indicating that it might play an important role in the pathogenesis of rickettsiae. However, in the absence of other evidence, its subcellular location of being surface-exposed was puzzling because ribosomal proteins are located in the cytoplasm. In the present study, the subcellular location of RpsB was analyzed with bioinformatics tools coupled with immunoelectron microscopy. The adhesion ability of RpsB was evaluated by protein microarray and cellular ELISA. Consequently, different bioinformatics tools gave different location predication results. Thus, RpsB was found in the cytoplasma and inner and outer membranes of* R. heilongjiangensis* by transmission electron microscopy. Protein microarray and cellular ELISA showed that RpsB binds to the host cell surface and its adhesion ability was even stronger than the known adhesin Adr1. In conclusion, RpsB was visually and directly shown for the time to be an SEP of rickettsiae and might be an important ligand and adhesin of rickettsiae. Its roles in pathogenesis warrant further study.

## 1. Introduction

*Rickettsia heilongjiangensis* is an obligate intracellular bacterium and is responsible for far-eastern spotted fever. It infects people through a tick bite and invades host cells through a “zipper-like” invasion mechanism [1], which is a receptor-ligand mediated mechanism. The ligand on the surface of rickettsiae stimulates the host cell receptor and subsequently induces series intracellular signal transduction pathways that recruit actin polymerization at the interaction sites of bacteria and the host cells, resulting in a membrane zipper construction around the bacteria. Based on this intracellular growth and “zipper-like” invasion mechanism, the surface-exposed proteins (SEPs) of rickettsiae play important roles in its pathogenesis, including rickettsial adherence to and invasion of host cells, intracellular bacterial growth and intercellular bacterial spread, and/or induction of the host-mediated immune responses against rickettsial infection.

Considering the important roles played by SEPs in the pathogenesis of rickettsiae, many studies have focused on identification of SEPs that are induced by rickettsiae, including those of* R. rickettsii *[2],* R. heilongjiangensis *[3],* R. conorii *[4],* R. felis *[5],* R. parkeri *[6],* Ehrlichia chaffeensis* [7],* Anaplasma phagocytophilum* [8], and* Coxiella burnetii *[9], using biotin-avidin affinity purification coupled with ESI-MS/MS. However among the identified SEPs in these studies, some were usually considered as cytoplasmic proteins, including 30S ribosomal protein S2 RpsB, 50S ribosomal protein L1 RplA, 50S ribosomal protein L25 RplY, and molecular chaperone GroEL. There are no signal peptides in their N- or C-terminal domains. Thus, it is puzzling to determine whether they were truly located on the surface of bacteria or just false positively identified. It is thus necessary to experimentally determine their subcellular location and the role that they play in disease pathogenesis.

In the present study, one of the ribosomal proteins, RpsB, was given further study. Its subcellular location in* R. heilongjiangensis* was confirmed by bioinformatics approaches and immunoelectron microscopy. In addition, its adhesion ability to host cells was evaluated by protein microarray assay and cellular ELISA.

## 2. Materials and Methods

### 2.1. Ethics Statement

Specific pathogen-free male BALB/c mice (4 to 6 weeks of age) were purchased from the Vital River Laboratories (Beijing, China). The animal experiments were approved by the local Administrative Committee for Laboratory Animals of Huadong Research Institute for Medicine and Biotechniques, and the animal care and husbandry met the standards of the committee. Mice were well cared for during their stay in the facility and all efforts were made to minimize suffering.

### 2.2. Bioinformatics Analysis of RpsB

The amino acid sequence of RpsB expressed by* R. heilongjiangensis* was searched from the National Center for Biotechnology Information (NCBI).

N-terminal signal peptides or nonclassical secretion signals (C-terminal signal peptides) in the protein were predicted by Signal-BLAST (http://sigpep.services.came.sbg.ac.at/signalblast.html) [10], SignalP 4.0 (http://www.cbs.dtu.dk/services/SignalP/) [11], LipoP 1.0 (http://www.cbs.dtu.dk/services/LipoP) [12], and SecretomeP 2.0 (http://www.cbs.dtu.dk/services/SecretomeP/) [13] servers. The subcellular location of RpsB was predicted by Gneg-mPLoc (http://www.csbio.sjtu.edu.cn/bioinf/Gneg-multi/) [14], SLP-Local (http://sunflower.kuicr.kyoto-u.ac.jp/~smatsuda/slplocal.html) [15], CELLO version 2 (http://cello.life.nctu.edu.tw/) [16], PSLpred (http://www.imtech.res.in/raghava/pslpred/submit.html) [17], SubLoc v1.0 [18], PSORTb 3.0.2 (http://www.psort.org/psortb/index.html) [19], and SOSUI-GramN (http://harrier.nagahama-i-bio.ac.jp/sosui/sosuigramn/sosuigramn_submit.html) [20] servers.

### 2.3. Preparation of Recombinant RpsB

Recombinant RpsB was expressed and purified as previously described [3]. Briefly, the nucleotide sequence of* rpsB* was amplified by the polymerase chain reaction (PCR) with its primer pairs (forward primer, 5′-GAGAATTCCATTTCGGTCACAAGA-3′; reverse primer, 5′-CTCTCGAGTAACGCCTTATCTGTATG-3′) and subsequently inserted into plasmid pET32a. The recombinant plasmid was transformed in* E. coli* and the expression of the recombinant protein was induced using IPTG. The resultant recombinant protein was purified by Ni-NTA affinity purification. Also the TrxA that was expressed by pET32a-transformed* E. coli* was purified in the same way as was done for the control. The purified proteins were analyzed by Western immunoblot and the concentration was determined using the Nano-drop 1000 method (Thermo Scientific, Hudson, NH, USA).

### 2.4. Preparation of Antisera against RpsB

Two groups of mice (three per group) were intraperitoneally immunized with 30 *μ*g of RpsB or TrxA that was mixed with complete Freund's adjuvant (Sigma-Aldrich) for primary immunization on day 0 and then injected with 20 *μ*g of the cognate antigen that was mixed with incomplete Freund's adjuvant (Sigma-Aldrich) on day 21. Mice were sacrificed on day 35 and the antisera were collected. Specific antibodies against RpsB or TrxA in the sera were analyzed by Western immunoblot [21]. To eliminate the influence of antibodies against proteins of* E. coli*, antisera against RpsB or TrxA were incubated with lysates of pET32a-transformed* E. coli* cells or nontransformed* E. coli*, respectively, for 1 h at 37°C and the precipitation was discarded by centrifugation just before the antigen-antibody reaction by Western immunoblotting.

### 2.5. Subcellular Location of RpsB by Immunoelectron Microscopy

Vero cells were cultured in Dulbecco's modification of Eagle's medium (DMEM) (Hyclone, Beijing, China) that was supplemented with 5% fetal bovine serum (FBS; Hyclone, San Jose, CA) and infected with* R. heilongjiangensis *[21]. For immunoelectron microscopy [2, 22], the* R. heilongjiangensis*-infected Vero cells were collected after 48 h of culture, and then fixed, dehydrated, and embedded in Spi-Pon 812 resin (Spi Supplies, West Chester, PA, USA) and transferred to a 200-mesh nickel grid (BeiJingZhongXingBaiRui Technology Co., Ltd., Beijing, China) as previously described [2]. The grids were then incubated with RpsB or TrxA immunized serum (1:10 dilution) for 2 h. After washing, the grids were incubated with goat anti-mouse IgG that was labeled with 10 nm colloidal gold particles (Aurion, EMS) (1:20 dilution) for 2 h, following which, they were washed, fixed in 1% glutaraldehyde for 10 min, stained with uranyl acetate (Spi Supplies, West Chester, PA) and lead citrate (Spi Supplies, West Chester, PA), and examined by transmission electron microscopy (TEM) at 80 kV (H-7650, Hitachi Chemical co., Ltd, Japan) [2].

### 2.6. Cy5 Labeling of Host Cell Protein

EAhy 926 cells were cultured in DMEM that was supplemented with 10% FBS and harvested after three days of culture. About 20 mg of cells were lysed and the whole cell protein was extracted using a protein extraction and labeling kit (Takara, Dalian, China). The obtained protein was labeled with a Cy5 fluorescent dye using the Cy5 monoreactive dye pack (GE Healthcare, Beijing, China) as per the manufacturer's instructions. The extra dye was removed using a PD-10 column (GE Healthcare) and the concentration of Cy5-labeled protein was determined by the Nano-drop 1000 method.

### 2.7. Protein Microarray Assay

The Adr1 (positive control, prepared as previously described [3]) or the TrxA (negative control) recombinant protein RpsB was printed onto polymer slides (Capitalbio, Beijing, China) as described previously [3], and each protein was printed as five replicate spots. The slide was blocked with a blocking buffer (PBS, 1% [w/v] BSA, pH 7.4) for 1 h. The Cy5-labeled cell protein was neutralized with* E. coli *lysates from cells that had been transformed with PET-32a plasmids for 2 h, following which, the supernatant was obtained after 10 min of centrifugation at 12 000 g. Next, the recombinant proteins on the blocked microarray were incubated with the neutralized Cy5-labeled cell protein for 1 h at room temperature. After three washes with PBS, the microarray was scanned with a GenePix Personal 4100A scanner (Molecular Devices, Sunnyvale, CA, USA) and the scanned images were analyzed by GenePix Pro 6.0 (Molecular Devices). The fluorescence intensity (FI) value of each protein was calculated by averaging the FI values of five replicate spots in which the backgrounds were subtracted [3].

### 2.8. Cellular ELISA

Each well of a 96-well plate was blocked with a blocking buffer at 4°C overnight, to prevent the proteins (RpsB, Adr1, or TrxA) from binding to the surface of the well. Next, the blocking buffer was removed and 100 *μ*l of EAhy 926 cells at a density of 1×10^5^/ml was added to each well. After 5 min of centrifugation at 800 g, the supernatant was removed, and 50 *μ*l of each recombinant protein (0.3 mg/ml of RpsB, Adr1, or TrxA) was added to four replicate wells. The recombinant proteins were incubated with host cells for 1 h at 37°C, after which, cells were washed three times in PBS. One hundred *μ*l of mouse anti-His-Tag antibody (1:2000 dilution; Raybiotech, Norcross, GA) was added and incubated for 1 h. After three washes in PBS, 100 *μ*l of a 1:5000 dilution of a horseradish peroxidase (HRP)-conjugated goat anti-mouse IgG (SBA, Birmingham, AL, USA) was added to each well for an additional one hour and incubated at 37°C. TMB ELISA substrate solution (eBioscience, San Diego, CA, USA) was added to each well for 5 min at room temperature. Thereafter, 50 *μ*l of H_2_SO_4_ (2 M) was added to stop the reaction. The optical density (OD) of each well was read at a wavelength of 450 nm using a standard microplate reader (UVM 340, ASYS HitechGmbH, Eugendorf, Austria) and the mean OD450 value of four replicate wells was calculated from this analysis.

### 2.9. Statistical Analysis

The results from each group were expressed as mean ± SD and analyzed by analysis of variance (ANOVA) or the Kruskal–Wallis Test according to their normal distribution and homogeneity of variance, followed by between-groups comparison by the Student–Newman–Keuls Test, using SAS version 9.1 statistical software (SAS Institute, Cary, NC, USA) [23].

## 3. Results

### 3.1. Bioinformatics Prediction

No signal peptides were identified for RpsB and TrxA as predicted by Signal-BLAST, SignalP, LipoP, or SecreteomeP database interrogation. It was predicted to be a cytoplasmic protein by SLP-Local, CELLO, and PSORTb, and an inner membrane protein by Gneg-mPLo, a periplasmic protein by PSLpred and SubLoc, and an extracellular protein by SOSUI-GramN database analyses, respectively.

### 3.2. Western Immunoblot Analysis of Immunized Sera

**Figure 1:**
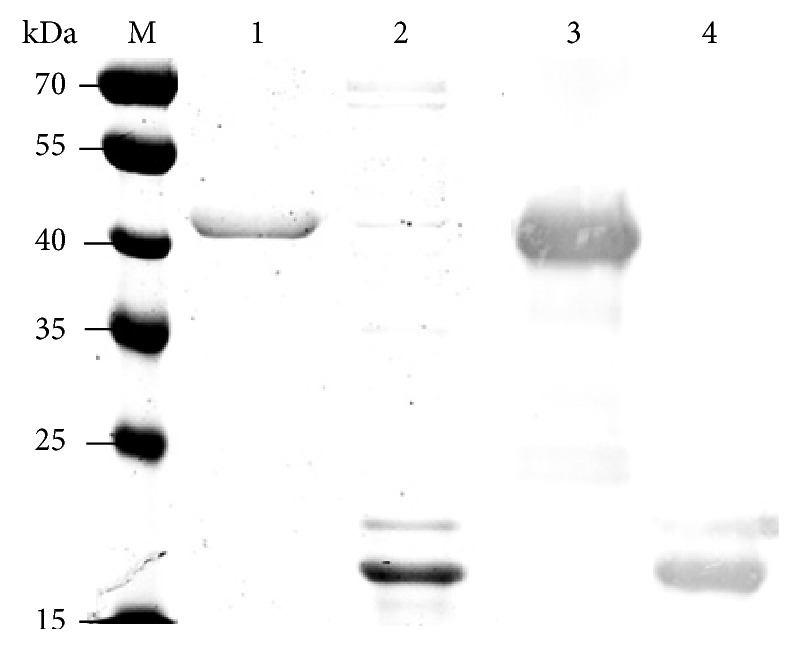
*Western blot analysis of specific antibodies against RpsB and TrxA in immunized mouse sera*. Purified RpsB (lane 1) and TrxA (lane 2) were analyzed by SDS-PAGE. Specific antibodies against RpsB (lane 3) and TrxA (lane 4) in immunized mouse sera were analyzed by Western immunoblotting. M: protein marker. The molecule size is indicated on the left.

The purified recombinant RpsB and TrxA (i.e., as a control protein) were used to immunize mice, and specific antibodies in the immunized sera were determined by Western immunoblot assay (Figure 1). Consequently, both RpsB and TrxA induced specific antibodies in immunized mouse sera, and the immunized sera were sufficient for capturing the corresponding protein.

### 3.3. Subcellular Location

**Figure 2:**
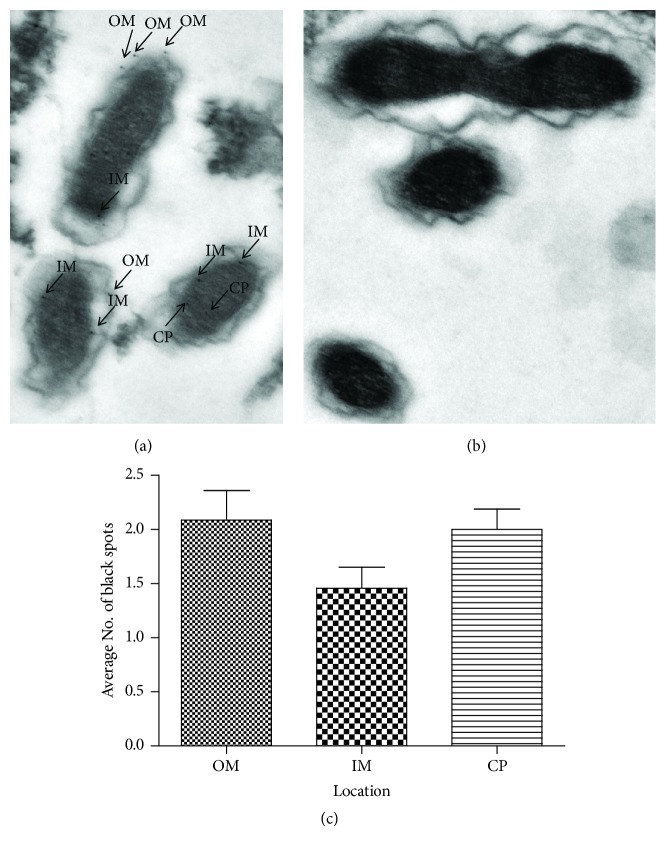
*Detection of RpsB in R. heilongjiangensis using immunoelectron microscopy*. Host cells that had been infected with* R. heilongjiangensis *were incubated with antisera against RpsB (a) and TrxA (b), and subsequently immunolabeled with colloidal gold particles (10 nm) using standard procedures. The cells were then observed by transmission electron microscopy. The average number of black spots of RpsB that were located on the inner membrane (im), on outer membrane (om), or in the cytoplasm (cp) were calculated (c).

The subcellular location of RpsB was analyzed by immunoelectron microscopy. As shown in Figure 2, the black spots indicated the location of the protein, and the number of spots indicated its relative abundance. RpsB was observed both on the inner/outer membranes and in the cytoplasm (Figure 2(a)). However, the control protein TrxA was not observed on the membrane nor in the cytoplasm of* R. heilongjiangensis* (Figure 2(b)), indicating that the binding of the antibody to RpsB in the bacteria was specific. Judging from the collected data of about 40 bacteria, we noted that more RpsB was observed on the outer membrane or cytoplasm than in the inner membrane, while the difference was not statistically significant by the Kruskal–Wallis Test (Figure 2(c)).

### 3.4. Protein Microarray Analysis

**Figure 3:**
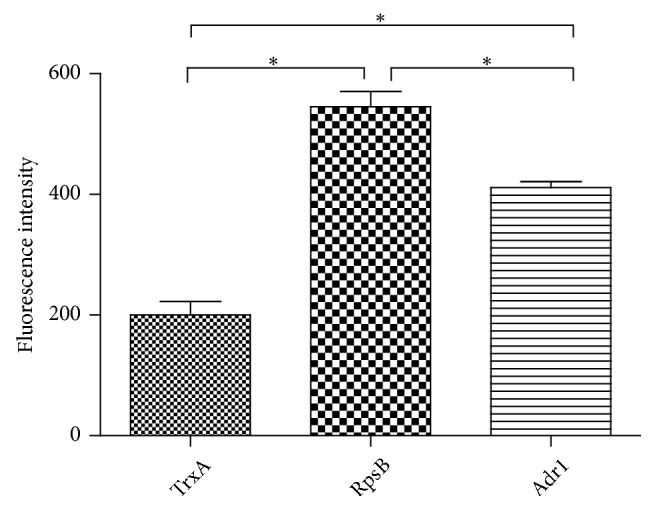
*Protein microarray analysis of recombinant RpsB with Cy5-labeled host cell proteins*. Adr1 and TrxA were used as positive and negative controls, respectively. A significant difference is indicated as ^*∗*^*P* < 0.05.

Protein microarray was used to analyze whether the recombinant protein could react with host cell proteins. A higher FI value indicated that more host cell proteins were captured by the indicated protein. As shown in Figure 3, the FI value of both RpsB and Adr1 (positive control) groups was significantly higher than that of the negative control TrxA group (i.e., over two-fold of TrxA;* P*<0.05), and the FI value of the RpsB group was significantly higher than that of the positive control Adr1 group (*P*<0.05), which indicated that both RpsB and Adr1 could react with host cell proteins and that RpsB exhibited a stronger binding capacity for host cell proteins than did Adr1.

### 3.5. Cellular ELISA

**Figure 4:**
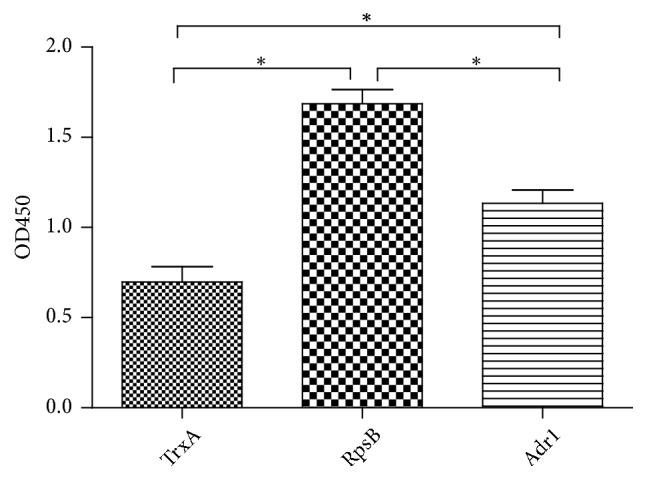
*Cellular ELISA of recombinant RpsB with EAhy 926 cells*. Adr1 and TrxA were used as positive and negative controls, respectively. Significant difference is indicated as ^*∗*^*P* < 0.05.

Cellular ELISA was used to analyze the adhesion ability of RpsB for the host cell surface. A higher OD450 indicated more proteins were captured by host cells. As shown in Figure 4, the OD450 of both the RpsB and Adr1 groups was significantly higher than that of the negative control TrxA group (i.e., 1.6- to 2.4-fold of TrxA;* P*<0.05), and the OD450 of RpsB was significantly higher than that of the positive control Adr1 (*P*<0.05), which indicated that RpsB exhibited a more potent adhesion ability with host cells than did Adr1.

## 4. Discussion

SEPs play important roles in the pathogenesis of intracellular parasites. In recent years, biotin-avidin affinity has been used to identify bacterial SEPs. Many SEPs were identified. However, some proteins were usually considered as cytoplasmic proteins and needed further confirmation.

RpsB is a ribosomal protein and is usually a cytoplasmic protein. It has been identified to be an SEP in both* R. felis *[5] and* R. heilongjiangensis* [3]. Further, it could react with sera from* R. heilongjiangensis*-infected mice or from patients and was considered a candidate diagnostic reagent [3, 24]. Considering the opposing views of subcellular location and the importance of this antigen, we confirmed its subcellular location by bioinformatics tools as well as by immunoelectron microscopy.

There is no signal peptide in RpsB. However, in the predictions of subcellular location, different bioinformatics tools gave varying results. This might be attributed to different predictive algorithms that are used by the selected tools. Also it might give us a hint that RpsB occupies several cellular locations. Moreover, observations made by immunoelectron microscopy confirmed this, wherein RpsB was observed to be predominantly located in the inner and outer membranes and was observed in the cytoplasma.

This is the first time that a ribosomal protein was confirmed to be surface-exposed in rickettsiae, and it solved our quest to identify its location. It also provides us more puzzles to solve as to whether other important ribosomal proteins, like RplA and RplY, are similarly distributed in the cell and whether RpsB was surface-exposed and to determine its role in disease pathogenesis.

To answer these questions, more studies are required. In this study, we preliminarily evaluated its ability to adhere to host cell proteins using protein microarray assay and cellular ELISA. Adr1 is a preidentified ligand and adhesin of spotted fever rickettsiae [25] and could bind to the surface of host cells. Thus, Adr1 was used as a positive control in this study. In the protein microarray assay, whole cell proteins were used as “prey” and recombinant proteins as “bait.” It is clear that both RpsB and Adr1 showed a more potent ability to capture host cell proteins than TrxA (Figure 3). To our surprise, RpsB captured more host cell protein than the positive control Adr1. However, the host cell protein captured by RpsB in the microarray plate might be a membrane or cytoplasmic protein. To confirm whether RpsB binds to the cell surface, cellular ELISA was conducted using intact host cells. Similar results to the microarray assay were found, indicating that RpsB binds to cell surface as did Adr1. The host cell proteins captured by RpsB in the microarray assay were membrane proteins. Additionally, RpsB exhibited a more potent adhesion to host cells than was observed for the positive control Adr1.

Actually, some other proteins that were traditionally considered cytoplasmic proteins were reported to be surface proteins in various pathogens. Bacterial elongation factor Tu was located on the surface of* Francisella tularensis* and plays an important role in bacterial adhesion and entry to host cells [26]. Another ribosomal protein L12 was reported to be a membrane-associated and surface-exposed protein in gonococci and functioned in gonococcal invasion of human reproductive cells [27, 28]. RpsB was shown to be exposed at the surface and in the periplasm of* Pseudomonas* [29, 30] and might be involved in the process of bacteriophage infection of* Pseudomonas aeruginosa* by interacting with the phage fiber [31]. Thus, it is not unexpected that RpsB is a surface-located protein that is important in the adhesion of* R. heilongjiangensis* to host cells.

In conclusion, the ribosomal protein RpsB is a seroreactive protein of* R. heilongjiangensis* that might be important in disease pathogenesis. In this study, the subcellular location was confirmed by bioinformatics coupled with immunoelectron microscopy. For the first time, RpsB was also visually and directly shown to be an SEP of rickettsiae. It displayed more potent adhesion to the host cell surface than Adr1 and might represent an important ligand and adhesin of rickettsiae. Its roles in the pathogenesis deserve further study.

## Data Availability

The data used to support the findings of this study are available from the corresponding author upon request.
